# Assessing the Efficacy of Protease Inactivation for the Preservation of Bioactive Amphibian Skin Peptides

**DOI:** 10.3390/ijms25168759

**Published:** 2024-08-12

**Authors:** Tatiana Yu. Samgina, Dmitrii M. Mazur, Albert T. Lebedev

**Affiliations:** 1 Department of Materials Science, MSU-BIT University, Shenzhen 517182, China; samguina@yandex.ru (T.Y.S.); neodmitrii@gmail.com (D.M.M.); 2 Department of Organic Chemistry, Lomonosov Moscow State University, Moscow 119991, Russia

**Keywords:** skin secretions of ranid frogs, bioactive peptides, peptides degradation, mass spectrometry, frogs’ proteases, proteolysis inhibitors

## Abstract

The skin of amphibians is a rich source of peptides with a wide range of biological activities. They are stored in secretory granules in an inactive form. Upon stimulation, they are secreted together with proteases into the skin. Once activated, they rapidly exert their biological effects, including fighting microorganisms and predators, while their excess is immediately destroyed by the released proteases. To keep bioactive peptides in their initial form, it is necessary to inhibit these enzymes. Several inhibitors for this purpose have previously been mentioned; however, there has not been any reliable comparison of their efficiency so far. Here, we studied the efficiency of methanol and hydrochloric and formic acids, as well as phenylmethylsulfonyl fluoride, in the inhibition of nine frog peptides with the known sequence, belonging to five families in the secretion of *Pelophylax esculentus*. The results demonstrated that methanol had the highest inhibitory efficiency, while phenylmethylsulfonyl fluoride was the least efficient, probably due to its instability in aqueous media. Possible cleavages between certain amino acid residues in the sequence were established for each of the inhibitors. These results may be helpful for future studies on the nature of proteases and on prediction of the possible cleavage sites in novel peptides.

## 1. Introduction

One of the most fascinating and prospective areas of modern science involves the study and application of natural products. Amphibian skin peptides are among these products due to their wide range of bioactivities [1,2]. Their study covers the problems of sequencing new peptides and establishing their biological properties. The first task nowadays mainly requires mass spectrometry [3], while novel benefits of this method appear regularly [4,5,6,7,8].

Amphibian skin peptides synthesized and stored in the dorsal glands, are part of their defense system against numerous pathogenic microorganisms, invasions, and predator attacks [9,10,11]. Amphibians have developed this protective arsenal through long evolution as a result of natural selection under the pressure of changing habitat conditions during migrations [12,13,14]. Along with alkaloids and biogenic amines, peptides constitute the majority of frog skin secretions. Since the pioneering work [15], which demonstrated the integral antimicrobial activity of *Rana ridibunda* secretion, the sequences of over two thousand peptides, united in more than 100 peptide families, have been established to date [10,16].

Ranid frogs (*Ranidae*) are not an exception, as peptides constitute the main part of their skin secretion [9]. Peptides secreted onto the skin are considered the first line of amphibian defense, as they possess a range of biological activities, such as antimicrobial, antiviral, antitumor, fungicidal, etc. [10,17,18,19]. They are capable of combating inflammatory processes and wound healing in the body of amphibians themselves, supporting the functioning of their skin as protective barriers [9,10,20,21]. All these issues enable treating frog skin peptides as prospective pharmaceuticals of future generations [22].

Simultaneously with peptides, alkaloids, and biogenic amines [9], amphibians secrete a set of proteases with various functions. Some of them may participate in the processes of post-translational modifications of peptides (PTM), such as the formation of N-terminal pyroglutamate, C-terminal amidation, sulfation and phosphorylation, formation of disulfide bonds, etc. [23,24,25,26,27,28]. Thus, C-terminal amidation of peptides [23] occurs under the action of peptidylglycine α-amidating monooxygenase, found in the skin of the clawed frog *Xenopus laevis* [23,24]. The site for amidation is the C-terminal Gly. Other peptidases participate in the processing of the mature peptides and their activation after secretion, e.g., by hydrolysis of the target peptide bonds with dipeptidyl aminopeptidase [24]. Processing peptidases can also participate in the degradation of secreted peptides [24]. Thus, the predominant degradation of peptide bonds Xaa-Lys by N-Lys, where Xaa is Leu, Gly, Ala, and Lys leads to the loss of activity of secreted peptides, and can be considered as a mechanism to prevent damage to the skin of amphibians [25], and the cysteine endopeptidase isolated from *X. laevis*, named after the amino acid in the active center, is capable of hydrolyzing the peptide to amino acids [26]. Amphibian secretions also contain peptidases that are more likely to recognize certain regions of the spatial configuration of peptides than their sequence. Thus, a specific endopeptidase, called magaininase, found in the secretion of *X. laevis*, breaks peptide bonds by binding to specific regions of the α-helical secondary structure of peptides [27,28]. Unfortunately, genomes of the Ranid frogs have not been studied so far. The nature of their enzymes remains unknown as well. Proteolytic degradation of peptides is initiated by amphibians rapidly: ~1–5 min after secretion, the activity of peptides and the secretion as a whole are no longer detected [29]. Bioactive peptide digestion by enzymes may play a certain positive role. Thus, the authors [30] demonstrated that bioactive cruzioseptines in vitro can prevent the emergence and dissemination of Listeria monocytogenes bacteria. At the same time, they may be digested by human trypsin. Therefore, these peptides are a potential food preservative harmless for humans.

Proteases, unlike amphibian peptides, are large protein molecules. Their precipitation can be achieved by changing the temperature, acidity of the solution, its ionic strength, etc. [31]. The latter is strictly necessary when one would like to study the sequence of bioactive peptides. The reagents used for this purpose must be inert with respect to the side chains of amino acids to preserve the intactness (nativeness) of the peptides. The solvents used should also be free of metal cations to prevent their introduction into the samples (critical when using buffer solutions or inorganic salts) so as not to complicate subsequent mass spectrometric experiments [32]. One of the traditionally used reagents for the precipitation of proteases is methanol, added to the skin secretion of amphibians after its washing with water in equal volume. An equal volume of methanol was determined experimentally, since at this ratio the degradation of peptides in the secretion practically ceased [33]. However, it was later shown that the added equal volume of methanol does not completely stop the process of peptide degradation in skin secretions: some proteases continue to retain their activity [29]. We have been using methanol in all our studies on the frogs’ peptides [34]. Nevertheless, other leading researchers in the field use other inhibitors in their similar studies. For example, Michael Conlon and Mauricio Simmaco apply acids [35,36,37,38,39,40,41]. The attractiveness of the latter involved their small introduced amount in comparison with methanol. Since the final concentration of acids in skin secretions should be only 1%, the final volume of the sample practically did not change upon the introduction of acids, while the use of methanol doubled it. Smaller volume reduced the time for purification, concentration, and lyophilization of the samples. Along with the listed reagents, the traditional inhibitor of serine proteases, phenylmethylsulfonyl fluoride (PMSF), is widely used in biochemistry for the inhibition, as most amphibian proteases belong to the serine type [28]. Its action differs from methanol or hydrochloric and formic acids: it does not affect the ionic strength of the solution but blocks the reactivity of proteases in secretions by selectively binding to their active center. In addition to chemical toxicity, a potential disadvantage of PMSF for our research objects may be its high instability in aqueous solutions, which are skin secretions, and insufficiently broad range of activity: PMSF is not active for all serine proteases.

Interestingly, nobody has yet compared the efficiency of the applied inhibitors. Therefore, in the current study, we decided to compare the most widespread inhibitors used for the protection of the integrity of the frog peptides to develop the most efficient procedure of sample preparation for future mass spectrometry sequencing. The aim of this work was to compare the effect of methanol and formic and hydrochloric acids, as well as PMSF, in preventing the degradation of peptides in the skin secretions of ranid amphibians and to find an optimal procedure to preserve bioactive peptides from degradation.

## 2. Results and Discussion

The inhibitory capacity of methanol and hydrochloric and formic acids, as well as PMSF at the minimum and maximum concentrations traditionally used in proteomics (0.1–1.0 mM), was compared via mass spectrometric monitoring of intact and proteolytic forms of nine peptides in five samples of *Pelophylax esculentus* secretions (samples 1–5). Table 1 shows the sequences of the peptides selected for monitoring established earlier [42,43,44]. 

The natural hybrid *P. esculentus* is formed by two parental species of green frogs (*P. ridibundus* and *P. lessonae*) [42,44]. All three species possess overlapping biometric parameters, which in practice makes it difficult to identify them by external features. Nevertheless, it is possible to reliably identify them by the composition of their skin peptidomes. It was shown that the skin secretions of the parental species of *P. esculentus* are individual in the composition of skin peptidomes, and their hybrid, as a rule, contains a set of peptides of two parental species. The letter extension in the name of the peptides in Table 1 indicates their belonging to the parental species (R—*P. ridibundus*; L—*P. lessonae*) [43]. All five individuals selected for this experiment were identified as individuals of the hybrid species *Pelophylax esculentus*, since their skin secretions contained peptides characteristic of its parental species (Table 1) [43]. 

All peptides in Table 1 are disulfide peptides, i.e., they contain an intramolecular S-S bond that forms a C-terminal loop (Rana box) [45]. Disulfide peptides with C-terminal OH-group were selected for the study as they demonstrate high antimicrobial activity while being at the same time the major components of the Ranid frogs’ secretion. They belong to four peptide families: esculentins 1 (No. 1, 2 in Table 1), having 46 amino acids in the sequences; brevinins 2 (No. 3–6), containing 29 or 33 amino acid links; brevinins 1 (No. 7, 8), with 24 amino acids in the chain; ranatuerins 2 (No. 9) with 17 amino acid links in the sequence. All disulfide peptides are usually membrane-active peptides and exhibit antimicrobial properties [9,10]. It is worth mentioning that we did not find any proteolytic fragments of the studied peptides being formed by the bond rupture inside the Rana box. This fact clearly demonstrates very high resistance of the cycle to any proteases released by frogs. 

Table 2 presents quantitative data for each of the nine peptides in Table 1, showing the percentage ratio of the intact (original) form of the peptide, the oxidized form (in the presence of methionine in the sequence), and the resulting proteolytic forms: products of the action of the remaining non-deactivated proteases. The full list of the detected proteolytic forms is included into the supporting material. The presence of one or another proteolytic form for each peptide in each of the five samples was determined by observing the coincidence of their exact experimental mass with the calculated one (within 0.5 ppm according to the mass spectra), as well as the coincidence of their sequences (tandem mass spectra data).

When calculating the data in Table 2, the total amount of intensities of all its formed forms in the mass spectra was taken as 100% for each peptide. To calculate the content in each of the samples of the intact, oxidized (if any), or proteolytic form of each of the nine peptides, the sum of the intensities of the chromatographic peaks of its multicharged ions, exactly corresponding to the values of the polyprotonated molecules of each of the forms, was used. The line “Proteoforms, %” took into account the sum of all proteolytic forms of a particular peptide for each of the five samples. The values of the intensities of each of the forms (intact, oxidized, and proteolytic) obtained in this way were summed up and, in relation to their resulting sum, the percentage content of each of the three forms in Table 2 was calculated for each of the five samples with different reagents.

According to Table 2, three of the nine peptides were present in the samples of secretions in the oxidized form, depending on the reagent. These were methionine-containing peptides, in which methionine was oxidized to methionine sulfoxide (esculentin 1, esculentin 1R, brevinin 1Ra). Methionine oxidation occurred in samples 1–4, except for sample 5, where the protease inhibitor PMSF was present in the maximum working concentration (1.0 mM). However, when the minimum concentration of PMSF was used, all three methionine-containing peptides were oxidized. Comparing samples 1–3, the lowest methionine oxidation occurred in the presence of methanol, where the only methionine-containing peptide, brevinin 1Ra, was oxidized.

Proteolytic forms of peptides in samples 1–5 were formed via hydrolysis of peptide bonds by proteases that remained active in the presence of the applied reagents. As a result, both N-terminal and C-terminal proteolytic forms were often present in the secretions. Table 2 reflects their total content (in %).

In general, according to Table 2, all peptides underwent the least degradation when methanol was used (sample 2): in its presence, the lowest quantity of proteolytic fragments was formed for all nine peptides, and the peptides themselves were preserved predominantly in their intact form, which indirectly indicated the most effective deactivation of the secreted proteases. Higher levels of proteolytic fragments in the experiments with acids definitely demonstrate lower inhibitory activity of these reagents. However, this feature may be treated as an advantage in some cases. Proteolytic fragments may be very useful for the de novo top-down sequencing of novel peptides [8], and their wider array or higher levels may be quite helpful, when sequence coverage of the initial peptide is low.

**Figure 1:**
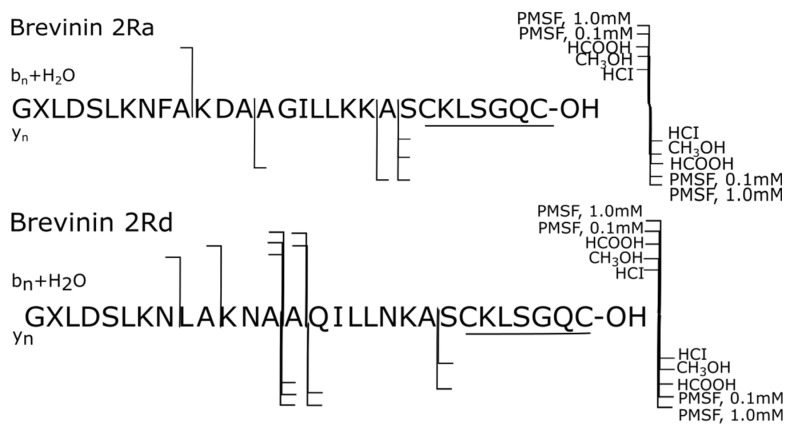
Degradation summary (samples 1–5): **top**—brevinin 2Ra; **bottom**—brevinin 2Rd.

The use of PMSF as a protease inhibitor for skin secretions of ranid amphibians was ineffective. In samples 4–5, the degradation processes of peptides prevailed (high values of the resulting proteolytic forms in samples 4, 5, Table 2) due to the rapid hydrolysis of PMSF in aqueous skin secretions, although the use of PMSF in the maximum possible working concentration (1.0 mM) prevented the oxidation of methionine in all three Met-containing peptides. The controversial data on the use of PMSF for all nine peptides in samples 4 and 5 (Table 2) indicate that not all proteases secreted by *P. esculentus* are serines. A 10-fold increase in the PMSF concentration does not affect the level of proteolytic forms formed: they continue to be formed in high and comparable amounts as in the experiment with the minimum working concentration of this inhibitor. The exception is brevinin 2Ra, in which there was a decrease in the formation of proteolytic forms by more than six times (from 88.21% to 14.01%), which is comparable to the order of increase in the working concentration of the inhibiting reagent in the secretion (samples 4 and 5). Only two proteolytic fragments formed by hydrolysis of the Ala^10^-Lys^11^ and Ala^13^-Ala^14^ bonds of brevinin 2Ra in the minimum amount of PMSF (sample 4) gave 88.21% of the sum of all the resulting proteolytic fragments: GXLDSLKNFA-OH (brevinin 2Ra (1–10)) and AGILLKKASCKLSGQC-OH (brevinin 2Ra (14–29)). When the concentration of the inhibitor of serine proteases was increased by 10 times, these breaks were no longer observed: the protease that caused them was deactivated (Figure 1). This fact may be indicative of the nature of the protease that caused these breaks: it is likely to be serine (PMSF is active against serine proteases).

Figure 1 top summarizes all the proteolytic forms of brevinin 2Ra that were formed in five samples in the presence of five reagents tested in the current research (listed on the right in Figure 1) and detected using LC-HRMS method and exact masses of the corresponding C- or N-terminal fragments.

Brevinin 2Ra did not degrade in the presence of methanol (Figure 1 top): there were no proteases left in the aqueous-methanol solution of Pelophylax esculentus skin secretion (sample 2) that were capable of hydrolyzing the peptide bonds of this molecule. In the presence of acids (samples 1 and 3), the only peptide bond Ala^21^-Ser^22^ was hydrolyzed with the formation of the C-terminal proteolytic fragment SCKLSGQC-OH (Figure 1 top). Structurally close brevinin 2Rd also did not degrade in the presence of methanol (Figure 1 bottom). Brevinin 2Rd has four substitutions in the sequence in comparison with brevinin 2Ra: Leu^9^→Phe^9^; Asn^12^→Asp^12^; Gln^15^→Gly^15^; Asn^19^→Lys^19^. In the presence of HCl, brevinin 2Rd formed a single C-terminal proteolytic fragment SCKLSGQC-OH due to the cleavage of the Ala^21^-Ser^22^ bond. The same proteolytic fragment was formed in the presence of HCl and HCOOH by brevinins 2Ra and 2L, since this conservative site and the Ala-Ser bond are present in their structures (samples 1 and 3).

**Figure 2:**
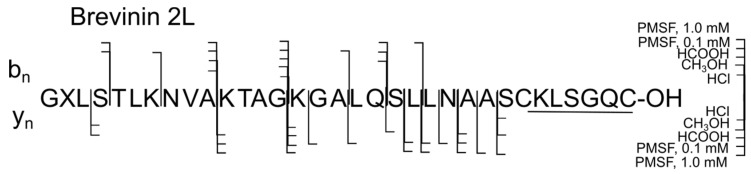
Degradation summary in brevinin 2L (samples 1–5). The proteolytic forms were detected using LC-HRMS method and exact masses of the corresponding C- or N-terminal fragments.

Brevinins 2L and 2La contain in the sequence 33 rather than 29 amino acids, and, with the exception of the C-terminal region, are not structurally close to brevinins 2Ra, 2Rd regions. Brevinin 2L contains three peptide bonds intersecting with brevinins 2Ra and 2Rd, which also degraded in samples 1–5. Thus, the Ala^10^-Lys^11^ bond degraded in all samples of the skin secretions, except for sample 1 containing HCl. The bond most susceptible to degradation in brevinin 2L is the peptide bond Gly^14^-Lys^15^: it degraded in the presence of all tested reagents (Figure 2) [25].

Brevinin 2La was present in the samples of Pelophylax esculentus secretion in vanishingly small amounts. In the sample with HCl, its intensive degradation occurred with the formation of two proteolytic forms K^26^CK(LV)GEC-OH and K^24^AKCK(LV)GEC-OH, the total share of which was 69.02% (hydrolysis of the bonds Asp^23^-Lys^24^ and Ala^25^-Lys^26^ correspondently). At the minimum concentration of PMSF and in the presence of HCOOH, two proteolytic fragments of brevinin 2La (1–10) and brevinine 2La (1–11) were formed, (hydrolysis of the bonds Gly^10^-Lys^11^ and Lys^11^-Ser^12^, respectively). In the presence of methanol and the maximum concentration of PMSF (1.0 mM), the degradation processes of this peptide were not observed at all, which may serve as indirect evidence of the serine nature of proteases that caused the ruptures Asp-Lys, Ala-Lys, Gly-Lys, and Lys-Ser in skin secretions (Supplementary Materials S1).

**Figure 3:**
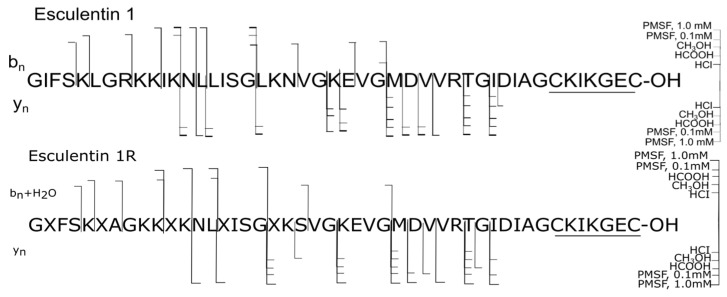
Degradation summary (samples 1–5): **top**—esculentin 1; **bottom**—esculentin 1R. The proteolytic forms were detected using LC-HRMS method and exact masses of the corresponding C- or N-terminal fragments.

The number of formed proteolytic forms, as shown above, directly depends on the amino acid sequence of peptides and their spatial structures, which they take in amphibian secretions. Esculentins 1 and 1R (46 aa), the longest of the analyzed peptides, were found to be most susceptible to degradation: they showed the highest percentage of formed proteolytic fragments of all peptides in Table 1 (samples 1–5) (Figure 3).

Esculentins 1R and 1 are structural analogs and have only three substitutions in the sequences: Gly^7^→Ala^7^; Arg^8^→Gly^8^; Asn^21^→Ser^21^. Degradation along the bonds Gly^27^-Met^28^ and Arg^32^-Thr^33^ in both esculentins occurred in the presence of all tested reagents, which failed to deactivate the proteases causing these ruptures, being apparently not serine (samples 1–5). The share of degradation products of both esculentins in the presence of all reagents was comparable, with the exception of the sample with HCl, where esculentin 1R formed half as many proteolytic forms: 27.69% versus 51.77% for esculentin 1. This decrease in degradation in the presence of HCl in esculentin 1R occurred due to the cessation of the formation of N-terminal proteolytic fragments (esculentin 1R (1–18), hydrolysis of the bond Gly^18^-Leu^19^), (esculentin 1R (1–27), hydrolysis of the bond Gly^27^-Met^28^), and C-terminal (esculentin 1R (35–46), hydrolysis of the bond Gly^34^-Ile^35^), whereas in esculentin 1, these processes continued to occur. That is, the presence of HCl stopped the hydrolysis of the bonds Gly-Xaa, where Xaa is Leu, Ile, and the bond Gly-Met with the formation of the C-terminal proteolytic fragment, while the formation of the N-terminal fragment of esculentin 1R (1–27) by hydrolysis of the same bond Gly-Met in the presence of HCl was preserved.

**Figure 4:**
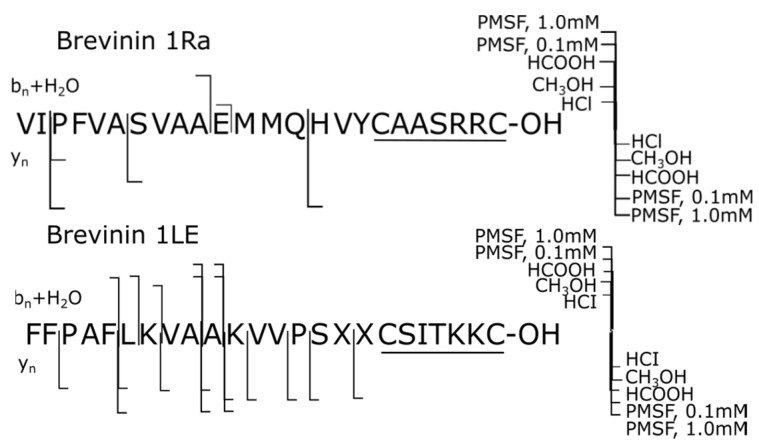
Degradation summary (samples 1–5): **top**—brevinin 1Ra 1; **bottom**—brevinin 1LE. The proteolytic forms were detected using LC-HRMS method and exact masses of the corresponding C- or N-terminal fragments.

The structures of brevinins 1Ra and 1LE in the presence of HCl, CH_3_OH, and HCOOH were the most resistant to degradation, i.e., the use of these reagents deactivated proteases capable of causing hydrolysis of the corresponding peptide bonds. Thus, in the presence of HCl and CH_3_OH, not a single proteolytic fragment was formed in brevinin 1LE and one fragment in brevinin 1Ra: this is the C-terminal brevinin 1Ra (3–24) and N-terminal brevinin 1Ra (1–11) with ruptures of the bonds Ile^2^-Pro^3^ and Glu^11^-Met^12^, respectively. The use of the serine protease inhibitor PMSF in both concentrations maintained this trend for brevinin 1Ra but led to an avalanche-like appearance of new degradation products in the structure of brevinin 1LE with intensive degradation along the bonds Phe^5^-Leu^6^, Leu^6^-Lys^7^, Ala^9^-Ala^10^, Ala^10^-Lys^11^, Val^13^-Pro^15^, and Pro^14^-Ser^15^ (Figure 4). 

The stability of brevinin 1Ra is possibly associated with the absence in its sequence of the bonds most susceptible to degradation: Xaa-Lys, where Xaa is Gly, Leu, Ala, and Lys, whereas in brevinin 1LE such bonds are present: Leu^6^-Lys^7^ and Ala^10^-Lys^11^. As already mentioned, in amphibian skin secretions, these peptide bonds are subject to intensive destruction by N-Lys [25]. Thus, the protease inhibitor PMSF in the range of working concentrations of 0.1–1.0 mM does not deactivate the proteases causing the listed ruptures of peptide bonds in brevinin 1LE.

**Figure 5:**
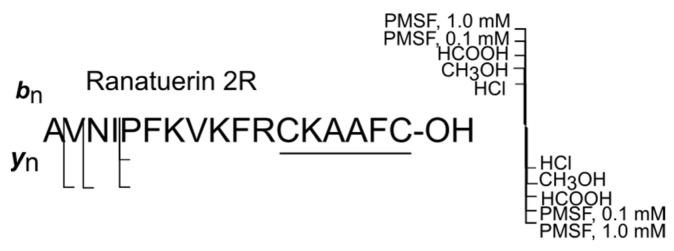
Degradation summary (samples 1–5) in ranatuerin 2R. The proteolytic forms were detected using LC-HRMS method and exact masses of the corresponding C- or N-terminal fragments.

Figure 5 shows the total degradation of ranatuerin 2R. As follows from Figure 5, ranatuerin 2R is extremely resistant to degradation. In the presence of methanol and PMSF in both concentrations (samples 2, 4, 5), its proteolytic fragments were not recorded. In the presence of HCl, a single fragment of ranatuerin 2R (5–17) was formed as a result of hydrolysis of the bond Ile^4^-Pro^5^, and three proteolysis products formed by cleavage of one, two, and four N-terminal amino acids: ranatuerin 2R (2–17), ranatuerin 2R (3–17), and ranatuerin 2R (5–17). This may be the result of the action of dipeptidylaminopeptidase, which has retained activity in the presence of hydrochloric and formic acids, and being capable of functioning as a processing enzyme, hydrolyzing Xaa-Pro bonds. In addition, ranatuerin 2R does not contain in the sequence any of the bonds most susceptible to destruction under the action of skin proteases: Xaa-Lys, where Xaa is Gly, Leu, Ala, and Lys [25].

The high resistance to degradation of ranatuerin 2R may also involve the fact that due to its high structural analogy with kunitzin-OS, an active trypsin inhibitor, it may also possess potential trypsin inhibitory activity [46,47]. Ranatuerin 2R has two substitutions in the structure in comparison with kunitzin-OS: Lys^9^Phe^10^→His^9^Leu^10^. Both ranatuerin 2R and ranatuerin 2Ra were discovered and sequenced by our group earlier in the secretion of *Pelophylax ridibundus* [42]. Their assignment to the ranatuerin 2 family was carried out according to the 6-membered disulfide cycle, which is a characteristic structural feature of ranatuerins 2. It was shown later that ranatuerin 2Ra was present in three species of green frogs that form the *P. esculentus complex*, while ranatuerin 2R was only in its parental species, *P. ridibundus* [43]. 28-membered peptides of this family with a characteristic 6-membered C-terminal cycle are present in the secretions of European populations of other ranid frogs, in particular *Rana arvalis* [5] and *Rana latastei* [48]. In the UniProtKB|Swiss-Prot database on the BLAST NCBI information resource, ranatuerin 2R and ranatuerin 2Ra were assigned ID P86019.1 and ID P86020.1, respectively. For some time, we believed that the detected 17-membered ranatuerins 2R and 2Ra were C-terminal degradation products of 28-membered ranatuerins, until a structurally similar peptide, 17-membered ishikawain 2, was isolated from the frog *Odorrana ishikawae*, whose complete sequence was confirmed by cDNAs cloning of encoding the precursor protein [49]:

A A I Y P F G I K I R CKAAFC—ishikawain 2

A V N I P F K V K F R CKAAFC—ranatuerin 2R

A A K I I L N P K F R CKAAFC—ranatuerin 2Ra

Mass spectrometric assignment of Ile^4^ in ranatuerin 2R was carried out in the paper published by Samgina et al. [42] The determination of Leu/Ile in the triad of isomeric amino acids of ranatuerin 2Ra was first made in 2012 [50] and secondarily confirmed in 2018 [7]. Since 2012, in works related to the complex of green frog *Pelophylax ecsulentus*, the sequence of ranatuerina 2Ra was indicated as AAKIILNPKFRCKAAFC-OH. The same peptide from the secretion of the same European species *Pelophylax ecsulentus* later received a second name kunitzin-RE [46]. Thus, the same peptide from one species appears now in the scientific literature under two names: ranatuerin 2Ra and kunitzin-RE.

## 3. Materials and Methods

### 3.1. Reagents

Methanol and acetonitrile were HPLC-gradient-grade, Sigma-Aldrich (St. Louis, MO, USA). Formic acid, also HPLC-gradient-grade, as well as dimethyl sulfoxide (DMSO) were commercially available (Fluka Buchs, Buchs, Switzerland); chemically pure HCl (Suprapur, Merck, Darmstadt, Germany) and HCOOH (Optima, LC-MS grade, Fisher Chemical, Mumbai, India) were commercially available. Water was prepared using the MilliQ water purification system (Millipore, Billerica, MA, USA).

### 3.2. Skin Secretions

The skin secretions of five sexually mature female specimens of the same size of *Pelophylax esculentus*, caught within the Moscow city limits, were obtained via mild electro-stimulation of the skin glands using a laboratory electro-stimulator [15,51]. The “milking” parameters were as follows: signal frequency 50 Hz, pulse duration 3 ms, voltage 12 V, total stimulation time 40 s. The secretion was washed off with deionized water into separate containers, to which the following reagents were immediately added individually: —HCl to final concentration 1% (sample 1); —CH_3_OH (sample 2); —HCOOH to final concentration 1% (sample 3); —0.1 mM PMSF (sample 4 in DMSO); —1.0 mM PMSF (sample 5 in DMSO). All samples were centrifuged for 15 min at 3 000 rpm, filtered through removable membrane filters (PTFE 0.45 μm), and lyophilized. The storage temperature was −26 °C. All five individuals were immediately returned to their place of capture after the procedure.

### 3.3. Compliance with Ethical Standards

All of the experiments with amphibians were carried out according to the rules published in Appendix A of the European Convention for the Protection of Vertebrate Animals Used for Experimental and Other Scientific Purposes (ETS No. 123), accepted in Strasbourg, 15 June 2006, and according to the Russian law GOST33219-2014, developed on the basis of the convention mentioned above by the Euro-Asian Council for Standardization, Metrology, and Certification (EASC).

### 3.4. Mass Spectrometry

Mass spectra were obtained on an Orbitrap ID-X high-resolution mass spectrometer (Thermo Scientific, Waltham, MA, USA) coupled to an LC-30 Nexera liquid chromatograph (Shimadzu, Kyoto, Japan) in LC-HRMS mode. An HPLC system consisted of two LC-30AD chromatographic pumps, a DGU-5A vacuum degasser, a SIL-30AC autosampler, an STO-20A column thermostat, and a CBM-20A system controller. The components of the secretions were separated on an XDB-C18 column (Agilent, Santa Clara, CA, USA) with a length of 100 mm and phase particle size of 1.8 μm. Gradient chromatography was performed using the following solutions: A—0.1% formic acid solution in deionized water (Milli-Q, Millipore, Molsheim, France); B—0.1% formic acid solution in acetonitrile containing 2% deionized water. All lyophilized samples were dissolved in phase B prior to the analysis. Injection volume was set to 0.1 µL. Elution was performed in a gradient of concentrations of solution B from 4% to 55% over 50 min, then from 55% to 90% over 5 min, kept at 90% of solution B for 2 min and decreased concentration of solution B back to 4% over 8 min, while solvent flow rate was established at 50 μL/min during the whole analysis.

Mass spectra were recorded in positive ion mode. For MS ions, the resolution was 120,000, and in MS/MS mode, 50,000. Collision-induced dissociation (CID) and higher-energy collision-induced dissociation (HCD) spectra were recorded in automatic mode. The details of experiments were as follows: inlet capillary voltage—3.5 kV, inlet capillary temperature—75 °C, and normalized cell energy (NCE) in CID and HCD mode were 28 and 35, correspondingly.

## 4. Conclusions

The disulfide-containing antimicrobial amphibian peptides become resistant to some extent to digestion by enzymes in the presence of all tested reagents. Methanol is the most efficient reagent for deactivating proteases in amphibian skin secretions and preservation of the intact peptides, despite the doubled volume of the resulting sample and partial oxidation of methionine, which is not an unsolvable problem for mass spectrometric sequencing. Nevertheless, as proteolytic fragments of the authentic peptides may be rather helpful in sequencing using mass spectrometry, hydrochloric and formic acids may also be treated as useful reagents. The use of PMSF when working with aqueous amphibian secretions is impractical due to its instability in aqueous solutions. Bonds that have more often undergone degradation in all solvents, taking into account all peptides, are Arg-Thr; Gly-Met, Gly-Ile, Gly-Lys; Gly-Leu. The most susceptible to degradation are long (46 aa) peptides from the esculentin 1 family: they form a high percentage of proteolytic fragments in the presence of all tested reagents, perhaps due to increased probability of finding the bonds most susceptible to hydrolysis in long peptides.

## Figures and Tables

**Table 1:** Skin peptides sequences of *Pelophylax esculentus* used for monitoring *.

№	Peptide	Mm, Da	Sequence
1	Esculentin 1	4882.7	GIFSKLGRKKIKNLLISGLKNVGKEVGMDVVRTGIDIAGCKIKGEC-OH
2	Esculentin 1R	4770.7	GXFSKXAGKKXKNLXISGXKSVGKEVGMDVVRTGIDIAGCKIKGEC-OH
3	Brevinin 2L	3242.8	GXLSTLKNVAKTAGKGALQSLLNAASCKLSGQC-OH
4	Brevinin 2La	3386.8	GXWNTXKATGKSA ASNVAVTLLDKAKCK(LV)GEC-OH
5	Brevinin 2Ra	2989.6	GXLDSLKNFAKDAAGILLKKASCKLSGQC-OH
6	Brevinin 2Rd	3011.6	GXLDSLKNLAKNAAQILLNKASCKLSGQC-OH
7	Brevinin 1Ra	2636.2	VIPFVASVAAEMMQHVYCAASRRC-OH
8	Brevinin 1LE	2607.5	FFPAFLKVAAKVVPSXXCSITKKC-OH
9	Ranatuerin 2R	1939.0	AVNIPFKVKFRCKAAFC-OH

* Underlined sequence shows C-terminal disulfide cycle.

**Table 2:** Percentage relation between three forms of peptides in samples 1–5 (intact, oxidized and proteolytic).

	Peptides *	Escul 1	Escul 1R	Brev 2L	Brev 2La	Brev 2Ra	Brev 2Rd	Brev 1Ra	Brev 1LE	Ranat 2R
Forms										
**HCl (sample 1)**										
Intact, %		38.61	64.86	98.79	30.98	98.98	99.61	93.51	100	85.21
Oxidized, %		9.63	7.45	-	-	-	-	5.74	-	-
Proteoforms, %		51.77	27.69	1.21	69.02	1.02	0.39	0.75	-	14.79
**CH_3_OH (sample 2)**										
Intact, %		79.99	77.85	95.34	100	100	100	98.27	100	100
Oxidized, %		-	-	-	-	-	-	1.13	-	-
Proteoforms, %		20.01	22.15	4.66	-	-	-	0.60	-	-
**HCOOH (sample 3)**										
Intact, %		72.22	84.41	95.26	95.02	98.70	97.77	99.51	98.37	65.01
Oxidized, %		10.79	-	-	-	-	-	0.19	-	-
Proteoforms, %		16.99	15.59	4.74	4.98	1.30	2.23	0.30	1.63	34.99
**PMSF (0.1 mM) (sample 4)**										
Intact, %		3.32	2.42	0.36	-	11.80	1.55	77.80	7.27	-
Oxidized, %		2.28	0.25	-	-	-	-	22.20	-	-
Proteoforms, %		94.40	97.33	99.64	-	88.21	98.45	-	92.73	-
**PMSF (1.0 mM) (sample 5)**										
Intact, %		8.10	0.74	19.49	-	85.99	20.84	99.47	10.26	100
Oxidized, %		-	-	-	-	-	-	-	-	-
Proteoforms, %		91.90	99.26	80.51	-	14.01	79.16	0.53	89.74	-

* The data involve peptides listed in Table 1.

## Data Availability

The data presented in this study are available for a limited time on request from the corresponding author.

## Supplementary Materials

The following supporting information can be downloaded at: https://www.mdpi.com/article/10.3390/ijms25168759/s1.
