# Supplementary material for: Assessing the Efficacy of Protease Inactivation for the Preservation of Bioactive Amphibian Skin Peptides
Source: Int J Mol Sci. 2024 Aug 12;25(16):8759. doi: 10.3390/ijms25168759 (PMC11354720; doi:10.3390/ijms25168759)
Supplement: Supplementary file 1 [file ijms-25-08759-s001.zip › ijms-3138022-supplementary.pdf]

Assessing the Efficacy of Protease Inactivation for the Pres

Tatiana Yu. Samgina [1,2], Dmitrii M. Mazur [1,2], Albert T. Lebedev[1,2]

[1] Department of Materials Science, MSU-BIT University, Shenzhen 517182, Ch
[2] Lomonosov Moscow State University, Department of Organic Chemistry, Mos

Supporting Information

m/z	Intensity	Charge	M+H	Peptide	RT, min
**Sample 1 HCl**					
485,7663	2477548	3,**4**,5	1940,0418	Ranatuerin-2R	22.7-27.6
817,4654	381929,2	**6**,7	4899,7534	Esculentin-1_Ox	27.6-31.8
811,6945	390363,9	**4**,5	3243,7546	Brevinin-2L	27.6-31.8
811,6946	22548390	3,**4**,5	3243,7550	**Brevinin-2L**	31.8-36.5
649,5566	873533,8	5	3243,7518	Brevinin-2L	36.5-41.4
649,557	112725,4	4,**5**	3243,7538	Brevinin-2L	46.4-52.0
664,074	608340,8	**4**,5	2653,2726	Brevinin-1Ra_Ox	27.6-31.8
664,0679	1495838	4	2653,2482	Brevinin-1Ra_Ox	31.8-36.5
664,0697	1284899	4	2653,2554	Brevinin-1Ra_Ox	36.5-41.4
664,07	64929,8	4	2653,2566	Brevinin-1Ra_Ox	41.4-46.5
611,3506	1531748	5,6,7,**8**,9	4883,7502	Esculentin-1	31.8-36.5
684,8182	473521,4	6,**7**	4787,6806	Esculentin-1R_Ox	31.8-36.5
796,1193	4122928	5,**6**,7,8,9	4771,6768	Esculentin-1R	31.8-36.5
678,3708	512991,1	5	3387,8228	Brevinin-2La	31.8-36.5
753,914	7965566	3,**4**,5	3012,6326	Brevinin-2Rd	36.5-41.4
753,9136	38634,4	4	3012,6310	Brevinin-2Rd	46.4-52.0
753,91	198033	**4**,5	3012,6166	Brevinin-2Rd	41.4-46.5
598,9294	3090792	4,**5**	2990,6158	Brevinin-2Ra	36.5-41.4
598,93	63678,7	5	2990,6188	Brevinin-2Ra	41.4-46.5
522,5017	17723618	3,4,**5**	2608,4773	Brevinin-1LE	36.5-41.4
522,5015	442999,8	3,4,**5**	2608,4763	Brevinin-1LE	46.4-52.0
660,0697	54745804	3,**4**	2637,2554	Brevinin-1Ra	36.5-41.4
660,07	1187736	3,**4**	2637,2566	Brevinin-1Ra	41.4-46.5
660,0693	325525,7	3,**4**	2637,2538	Brevinin-1Ra	46.4-52.0
**Proteoforms**					
412,1756	32394	2	823,3434	Brevinin-2L, Brevinin-2	13.5-18.5
416,5424	437771,4	3	1247,6116	Esculentin-1, Esculenti	18.5-22.75
359,5261	238062,2	3	1076,5627	Brevinin-2La	18.5-22.75
359,5261	904965,8	3	1076,5627	Brevinin-2La	22.7-27.6
514,9406	430075,2	3	1542,8062	Ranatuerin-2R	22.7-27.6
469,2322	922478,1	3	1405,6810	Esculentin-1R, Esculen	22.7-27.6
458,277	260780	3	1372,8154	Brevinin-2L	22.7-27.6
368,2104	450140,8	3	1102,6156	Brevinin-1Ra	22.7-27.6
580,1628	293657,3	5	2896,7828	Esculentin-1	27.6-31.8
502,2526	400016	4	2005,9870	Esculentin-1, Esculenti	27.6-31.8
495,8028	323969,1	2	990,5978	Esculentin-2LE	27.6-31.8

Sample 2 CH3OH

485,7663	185806,2	4	1940,0418	Ranatuerin-2R	23.51-28.27
668,0668	53413,9	4	2669,2438	Brevinin-1Ra_2Ox	28.05-32.07
664,0677	166164,1	4	2653,2474	Brevinin-1Ra_Ox	34.76-39.27
814,7987	915301,1	5,**6**,7,8,9	4883,7532	Esculentin-1	31.97-34.82
814,7972	123192	**6**,7	4883,7442	Esculentin-1	34.76-39.27
796,1195	320533,2	5,**6**,7,8	4771,6780	Esculentin-1R	31.97-34.82
796,1189	1003724	5,**6**,7,8,9	4771,6744	Esculentin-1R	34.76-39.27
796,1183	11728,1	6	4771,6708	Esculentin-1R	43.94-49.03
678,3706	60650,7	5	3387,8218	Brevinin-2La	31.97-34.82
678,3704	96391,4	5	3387,8208	Brevinin-2La	34.76-39.27
649,5574	6464500	3,4,**5**	3243,7558	Brevinin-2L	34.76-39.27
649,5566	198953	5	3243,7518	Brevinin-2L	39.06-43.96
649,5568	39026,4	4,**5**	3243,7528	Brevinin-2L	43.94-49.03
649,5567	20738,4	4,**5**	3243,7523	Brevinin-2L	48.95-55.11
522,5008	86157,4	4,**5**	2608,4728	Brevinin-1LE	34.76-39.27
522,5013	1063591	4,**5**	2608,4753	Brevinin-1LE	39.06-43.96
522,5016	1078636	3,4,**5**	2608,4768	Brevinin-1LE	43.94-49.03
522,5014	389790	3,4,**5**	2608,4758	Brevinin-1LE	48.95-55.11
753,9133	1244868	3,**4**,5	3012,6298	Brevinin-2Rd	39.06-43.96
753,9134	15839,8	4	3012,6302	Brevinin-2Rd	43.94-49.03
753,9135	5794,4	4	3012,6306	Brevinin-2Rd	48.95-55.11
598,9289	434176	**4**,5	2990,6133	Brevinin-2Ra	39.06-43.96
660,0696	18850700	3,**4**	2637,2550	Brevinin-1Ra	39.06-43.96
660,0689	243825,5	3,**4**	2637,2522	Brevinin-1Ra	43.94-49.03
660,069	97825,1	3,**4**	2637,2526	Brevinin-1Ra	48.95-55.11
Proteoforms					
469,2325	68810,3	3	1405,6819	Esculentin-1, Esculenti	19.65-23.47
416,5424	59053,8	2,**3**	1247,6116	Esculentin-1, Esculenti	19.65-23.47
567,7676	35557,6	2	1134,5274	Esculentin-1, Esculenti	19.65-23.47
502,2526	52687,7	4	2005,9870	Esculentin-1, Esculenti	23.51-28.27
458,277	67526,9	3	1372,8154	Brevinin-2L	23.51-28.27
359,8692	50449,9	3	1077,5920	Brevinin-2Lb, Brevinin	23.51-28.27
592,9189	74214,3	**4**,5	2960,5633	Brevinin-2L	28.05-32.07
581,509	68680	5	2903,5138	Esculentin-1R	28.05-32.07
666,3394	51682,5	4	2662,3342	Esculentin-1R	28.05-32.07
605,5591	43665,9	4	2419,2130	Esculentin-1, Esculenti	28.05-32.07
453,9501	36134,4	3	1359,8347	Esculentin-2LE	28.05-32.07
495,8028	100241,3	2	990,5978	Esculentin-2LE	28.05-32.07
630,657	187061,5	3	1889,9554	Brevinin-2L	31.97-34.82
809,0388	118764	3	2425,1008	Brevinin-1Ra	39.06-43.96

Sample 3 HCOOH

485,768	92309,7	4	1940,0486	Ranatuerin-2R	18.91-24.80
388,8141	367291,2	3,4,**5**	1940,0393	Ranatuerin-2R	24.56-28.64
668,0667	51746	4	2669,2434	Brevinin-1Ra_2Ox	28.56-31.77
814,7987	1742701	5,**6**,7,8,9	4883,7532	Esculentin-1	31.82-35.70
796,1191	1494774	5,**6**,7,8,9	4771,6756	Esculentin-1R	31.82-35.70
796,1189	569116,7	**6**,7,8	4771,6744	Esculentin-1R	35.67-38.91

678,3706	323897,4	5	3387,8218	Brevinin-2La	31.82-35.70
649,5574	13692233	3,4,**5**	3243,7558	Brevinin-2L	31.82-35.70
649,5573	1149257	3,4,**5**	3243,7553	Brevinin-2L	35.67-38.91
649,5567	287523,4	4,**5**	3243,7523	Brevinin-2L	38.82-42.52
811,6937	90327,6	**4**,5	3243,7514	Brevinin-2L	42.52-47.00
649,5568	41496,4	4,**5**	3243,7528	Brevinin-2L	46.95-55.11
522,5009	336517	4,**5**	2608,4733	Brevinin-1LE	35.67-38.91
522,5015	8745915	3,4,**5**	2608,4763	Brevinin-1LE	38.82-42.52
522,5013	4031055	3,4,**5**	2608,4753	Brevinin-1LE	42.52-47.00
522,5014	558160,4	3,4,**5**	2608,4758	Brevinin-1LE	46.95-55.11
753,914	8543052	3,**4**,5	3012,6326	Brevinin-2Rd	38.82-42.52
753,9132	175975,2	**4**,5	3012,6294	Brevinin-2Rd	42.52-47.00
753,9134	34655,6	4,**5**	3012,6302	Brevinin-2Rd	46.95-55.11
598,9294	3477826	3,4,**5**,6	2990,6158	Brevinin-2Ra	38.82-42.52
598,9289	66343,5	5	2990,6133	Brevinin-2Ra	42.52-47.00
598,929	16098,4	5	2990,6138	Brevinin-2Ra	46.95-55.11
660,0698	26451080	3,**4**	2637,2558	Brevinin-1Ra	38.82-42.52
660,0688	437845,6	3,**4**	2637,2518	Brevinin-1Ra	42.52-47.00
660,0691	115458	3,**4**	2637,2530	Brevinin-1Ra	46.95-55.11
Proteoforms					
388,5116	6987,6	3	1163,5192	Brevinin-1Ra	13.89-19.00
483,2127	18320,7	2	965,4176	Brevinin-2L	13.89-19.00
412,1755	8067,4	2	823,3432	Brevinin-2L, Brevinin-2	13.89-19.00
469,2321	91279,8	3	1405,6807	Esculentin-1, Esculenti	18.91-24.80
416,5423	39437,8	3	1247,6113	Esculentin-1, Esculenti	18.91-24.80
580,1623	28647,1	5	2896,7803	Esculentin-1	24.56-28.64
580,1625	33847,6	5	2896,7813	Esculentin-1	28.56-31.77
548,9443	43543,5	**5**,6	2740,6903	Esculentin-1	24.56-28.64
582,6218	33954,3	4	2327,4638	Esculentin-1	24.56-28.64
554,603	37070,3	**4**,5	2215,3886	Esculentin-1R	24.56-28.64
669,3338	55438,9	**3**,4	2005,9858	Esculentin-1,Esculentin	24.56-28.64
493,8163	15761,4	4	1972,2418	Esculentin-1	24.56-28.64
468,2836	23100,7	3,**4**	1870,1110	Brevinin-2L	24.56-28.64
468,0062	59563,5	4	1869,0014	Ranatuerin-2R	24.56-28.64
608,3161	26323,1	3	1822,9327	Brevinin-2Lb	24.56-28.64
590,6495	27599,3	3	1769,9329	Ranatuerin-2R	24.56-28.64
439,1904	18666,9	4	1753,7382	Brevinin-1Ra	24.56-28.64
558,9604	98882,7	3	1674,8656	Brevinin-2Rd	24.56-28.64
535,2811	13343,7	3	1603,8277	Brevinin-2Rd	24.56-28.64
514,9407	160225,7	3	1542,8065	Ranatuerin-2R	24.56-28.64
696,8341	26774,3	2	1392,6604	Brevinin-2L	24.56-28.64
458,2771	104523,7	2,**3**	1372,8157	Brevinin-2L	24.56-28.64
396,8954	16967,5	3	1188,6706	Brevinin-2La	24.56-28.64
508,311	68952,7	2	1015,6142	Brevinin-2L	24.56-28.64
430,2479	46793	2	859,4880	Brevinin-2Rd, Brevinin	24.56-28.64
423,224	14764,9	1	423,2240	Esculentin-1, Esculenti	24.56-28.64
592,9189	47740,1	**4**,5	2960,5633	Brevinin-2L	28.56-31.77
581,5087	58339,1	5	2903,5123	Esculentin-1R	28.56-31.77
557,747	32216,3	5	2784,7038	Esculentin-1R	28.56-31.77
605,559	52605,6	4	2419,2126	Esculentin-1, Esculenti	28.56-31.77

562,5452	39565,1	4	2247,1574	Brevinin-2L	28.56-31.77
503,4746	56037,5	4	2010,8750	Brevinin-1Ra	28.56-31.77
500,5505	62041,9	4	1999,1786	Brevinin-1LE	28.56-31.77
440,2559	66861,3	4	1758,0002	Brevinin-1LE	28.56-31.77
464,5891	37745	3	1391,7517	Brevinin-2Lb, Brevinin	28.56-31.77
678,8957	41001,9	2	1356,7836	Brevinin-2Rd	28.56-31.77
495,8028	90649,5	2	990,5978	Esculentin-2LE	28.56-31.77
435,2496	55917,8	2	869,4914	Brevinin-1LE	28.56-31.77
630,657	214392,5	3	1889,9554	Brevinin-2L	31.82-35.70
773,4026	260524,3	4	3090,5870	Esculentin-1_Ox	38.82-42.52
579,3402	41966,2	4	2314,3374	Brevinin-1LE	42.52-47.00

Sample 4 PMSF 0.1 mM

698,5424	54490,3	6,**7**	4883,7500	Esculentin-1	30.08-35.02
698,5422	42679,2	6,**7**,8	4883,7486	Esculentin-1	35.00-40.03
698,5419	6645,8	6,**7**	4883,7465	Esculentin-1	39.98-45.19
798,7868	9047,9	6	4787,6818	Esculentin-1R_Ox	35.00-40.03
796,1188	72488,9	5,**6**,7,8	4771,6738	Esculentin-1R	35.00-40.03
796,1185	15376,9	6	4771,6720	Esculentin-1R	39.98-45.19
649,5568	57365,5	4,**5**	3243,7528	Brevinin-2L	35.00-40.03
649,5565	6336,1	5	3243,7513	Brevinin-2L	39.98-45.19
664,0684	130365,9	3,**4**	2653,2502	Brevinin-1Ra_Ox	35.00-40.03
664,068	12804,2	4	2653,2486	Brevinin-1Ra_Ox	39.98-45.19
660,0693	35888,1	4	2637,2538	Brevinin-1Ra	35.00-40.03
660,0696	397435,6	3,**4**	2637,2550	Brevinin-1Ra	39.98-45.19
660,0691	53011,1	3,**4**	2637,2530	Brevinin-1Ra	45.32-50.18
660,0691	15475,5	3,**4**	2637,2530	Brevinin-1Ra	50.10-55.16
522,5009	24746	4,**5**	2608,4733	Brevinin-1LE	35.00-40.03
522,5009	61118,6	4,**5**	2608,4733	Brevinin-1LE	39.98-45.19
522,5009	101199,9	3,4,**5**	2608,4733	Brevinin-1LE	45.32-50.18
522,5008	77062,2	3,4,**5**	2608,4728	Brevinin-1LE	50.10-55.16
753,9136	26097,7	**4**,5	3012,6310	Brevinin-2Rd	39.98-45.19
598,929	13194	5	2990,6138	Brevinin-2Ra	39.98-45.19

Proteoforms

706,3807	24923,6	5	3527,8723	Esculentin-1	35.00-40.03
660,9552	67007,1	5	3300,7448	Esculentin-1	30.08-35.02
655,5532	167483,5	5	3273,7348	Esculentin-1R	30.08-35.02
590,1098	71294,5	5	2946,5178	Esculentin-1_Ox	25.59-30.11
586,9112	1480191	4,**5**,6	2930,5248	Esculentin-1	25.59-30.11
586,9107	133377	5	2930,5223	Esculentin-1	30.08-35.02
581,5092	1985065	5	2903,5148	Esculentin-1R	25.59-30.11
581,5087	9359,2	5	2903,5123	Esculentin-1R	35.00-40.03
726,6339	49286,4	4	2903,5122	Esculentin-1R	30.08-35.02
605,5592	100369,5	4	2419,2134	Esculentin-1, Esculenti	25.59-30.11
562,5455	320106,3	3,**4**	2247,1586	Brevinin-2L	30.08-35.02
502,2528	496311,3	3,**4**	2005,9878	Esculentin-1, Esculenti	25.59-30.11
493,8163	60902,4	4	1972,2418	Esculentin-1	21.00-25.70
630,6573	7251661	3	1889,9563	Brevinin-2L	30.08-35.02
630,6572	59954,9	3	1889,9560	Brevinin-2L	35.00-40.03

630,6571	21519,9	3	1889,9557	Brevinin-2L	45.32-50.18
630,6571	7812,3	3	1889,9557	Brevinin-2L	50.10-55.16
625,6533	66997,1	3	1874,9443	Esculentin-1, Esculenti	25.59-30.11
468,2834	71821,7	3,**4**	1870,1102	Brevinin-2L	25.59-30.11
881,4348	52585,5	2	1761,8618	Brevinin-2L	30.08-35.02
440,7356	36371,6	4	1759,9190	Esculentin-1, Esculenti	21.00-25.70
558,9607	887998,5	2,**3**	1674,8665	Brevinin-2Rd	21.00-25.70
558,9604	92728,8	3	1674,8656	Brevinin-2Rd	25.59-30.11
817,4053	229901,3	2	1633,8028	Brevinin-2L	25.59-30.11
543,6611	66601,5	3	1628,9677	Brevinin-2L	25.59-30.11
539,9652	98640,6	**3**,4	1617,8800	Brevinin-2Ra	21.00-25.70
802,4185	36241,2	**2**,3	1603,8292	Brevinin-2Rd	21.00-25.70
529,9701	389706	**3**,4	1587,8947	Brevinin-1LE	25.59-30.11
379,9949	31005,3	4	1516,9562	Esculentin-1R	21.00-25.70
506,2911	659757,4	**3**,4	1516,8577	Brevinin-1LE	25.59-30.11
714,4146	102357,8	**2**,3	1427,8214	Brevinin-2Rd	30.08-35.02
469,2327	143127,3	3	1405,6825	Esculentin-1, Esculenti	21.00-25.70
464,5892	114080	3	1391,7520	Brevinin-2Lb, Brevinin	30.08-35.02
463,5928	58728,9	3	1388,7628	Brevinin-1LE	30.08-35.02
344,479	6905,1	4	1374,8926	Esculentin-1	17.78-21.01
458,277	6101544	2,**3**	1372,8154	Brevinin-2L	25.59-30.11
458,2767	24302,9	3	1372,8145	Brevinin-2L	35.00-40.03
453,95	417656,6	2,**3**	1359,8344	Esculentin-2LE	30.08-35.02
452,9332	473607,2	2,**3**	1356,7840	Brevinin-2Rd	30.08-35.02
653,318	315754,7	2	1305,6282	Brevinin-2L	21.00-25.70
416,5424	32719,8	3	1247,6116	Esculentin-1, Esculenti	21.00-25.70
596,776	71468,4	2	1192,5442	Brevinin-2L	21.00-25.70
595,8169	125743	2	1190,6260	Brevinin-1LE	25.59-30.11
373,5694	585291,6	2,**3**	1118,6926	Esculentin-2LE	25.59-30.11
555,8209	996703	2	1110,6340	Brevinin-1LE	30.08-35.02
555,8209	18483,7	2	1110,6340	Brevinin-1LE	35.00-40.03
547,2908	112602,1	2	1093,5738	Brevinin-1LE	25.59-30.11
540,234	25609,9	2	1079,4602	Brevinin-2L	17.78-21.01
359,8691	32388,6	3	1077,5917	Brevinin-2Lb, Brevinin	21.00-25.70
530,7922	163643,1	**2**,3	1060,5766	Brevinin-2La	25.59-30.11
350,222	69341,8	3	1048,6504	Esculentin-1R	21.00-25.70
522,3083	68786,8	2	1043,6088	Brevinin-2Rd	30.08-35.02
520,3024	712789,6	2	1039,5970	Brevinin-1LE	30.08-35.02
520,3024	5990,8	2	1039,5970	Brevinin-1LE	39.98-45.19
508,3112	18357,4	2	1015,6146	Brevinin-2L	35.00-40.03
508,3109	2439736	2	1015,6140	Brevinin-2L	25.59-30.11
495,8029	17985,7	2	990,5980	Esculentin-2LE	39.98-45.19
495,8028	3981059	2	990,5978	Esculentin-2LE	25.59-30.11
495,8028	27568,8	2	990,5978	Esculentin-2LE	35.00-40.03
495,8027	446941,5	2	990,5976	Esculentin-2LE	30.08-35.02
483,2126	20851,8	2	965,4174	Brevinin-2L	17.78-21.01
447,2328	66468,5	2	893,4578	Brevinin-1LE	25.59-30.11
293,18	81873,9	3	877,5244	Esculentin-1	21.00-25.70
410,7501	72158,6	2	820,4924	Esculentin-2LE	25.59-30.11
382,2394	68647,2	2	763,4710	Esculentin-2LE	25.59-30.11

741,3972	52992,1	1	741,3972	Brevinin-1LE	35.00-40.03
368,2237	132592,1	2	735,4396	Esculentin-1R	25.59-30.11
366,2368	41099,6	2	731,4658	Brevinin-2L	21.00-25.70
628,313	169623,8	1	628,3130	Brevinin-1LE	30.08-35.02
276,1631	219805,4	2	551,3184	Esculentin-1, Esculenti	21.00-25.70
389,2395	351206,4	1	389,2395	Brevinin-2L	17.78-21.01

Sample 5 PMSF 1.0 mM

388,8137	80780,4	5	1940,0373	Ranatuerin-2R	25.52-31.07
814,7984	337464	**6**,7,8	4883,7514	Esculentin-1	31.00-34.74
698,542	117124,2	6,**7**,8	4883,7472	Esculentin-1	34.67-39.47
796,1187	60137,2	**6**,7	4771,6732	Esculentin-1R	34.67-39.47
649,5574	3780648	3,4,**5**	3243,7558	Brevinin-2L	34.67-39.47
649,5566	86446,8	4,**5**	3243,7558	Brevinin-2L	39.32-45.24
649,5567	21232,8	4,**5**	3243,7523	Brevinin-2L	45.22-49.98
603,3322	130215,3	3,4,**5**	3012,6298	Brevinin-2Rd	39.32-45.24
598,9289	140365,2	4,**5**	2990,6133	Brevinin-2Ra	39.32-45.24
660,0697	7302195	2,3,**4**	2637,2554	Brevinin-1Ra	39.32-45.24
660,0695	315701,2	3,**4**	2637,2546	Brevinin-1Ra	45.22-49.98
522,5008	34015,5	4,**5**	2608,4728	Brevinin-1LE	39.32-45.24
522,5009	13901,5	4,**5**	2608,4733	Brevinin-1LE	45.22-49.98

Proteoforms

706,3806	112179,7	5	3527,8718	Esculentin-1	34.67-39.47
700,9782	55912,4	5	3500,8598	Esculentin-1R	34.67-39.47
683,5721	103579,5	5	3413,8293	Esculentin-1	31.00-34.74
660,9551	216819,2	5	3300,7443	Esculentin-1	31.00-34.74
655,5536	574841,1	4,**5**,6	3273,7368	Esculentin-1R	31.00-34.74
586,9114	2160776	3,4,**5**,6	2930,5258	Esculentin-1	25.52-31.07
581,5093	3611419	3,4,**5**,6	2903,5153	Esculentin-1R	25.52-31.07
581,5084	83364,5	5	2903,5108	Esculentin-1R	31.00-34.74
607,0313	29982,4	3,**4**	2425,1018	Brevinin-1Ra	39.32-45.24
605,559	237642,5	**4**,5	2419,2126	Esculentin-1, Esculenti	25.52-31.07
562,5458	635039,4	3,**4**	2247,1598	Brevinin-2L	31.00-34.74
690,7471	240065,9	**3**,4	2070,2257	Brevinin-2L	25.52-31.07
502,2527	533330,3	**3**,4	2005,9874	Esculentin-1, Esculenti	25.52-31.07
500,5505	126192,4	4	1999,1786	Brevinin-1LE	25.52-31.07
493,8171	1013209	3,**4**,5	1972,2450	Esculentin-1	21.09-25.67
493,8166	240774,8	**4**,5	1972,2430	Esculentin-1	25.52-31.07
630,6572	9506916	2,**3**	1889,9560	Brevinin-2L	31.00-34.74
630,6572	22550,3	3	1889,9560	Brevinin-2L	45.22-49.98
630,6571	101150,8	3	1889,9557	Brevinin-2L	34.67-39.47
630,657	49494,6	3	1889,9554	Brevinin-2L	39.32-45.24
472,5508	1773132	4	1887,1798	Esculentin-1R	25.52-31.07
625,6533	99347,7	3	1874,9443	Esculentin-1, Esculenti	25.52-31.07
468,2838	98101,3	4	1870,1118	Brevinin-2L	25.52-31.07
440,7353	101568,6	4	1759,9178	Esculentin-1,Esculenti	21.09-25.67
558,9604	128460,8	3	1674,8656	Brevinin-2Rd	21.09-25.67
558,9604	200948,7	3	1674,8656	Brevinin-2Rd	25.52-31.07
401,261	115015,1	4	1602,0206	Esculentin-1	21.09-25.67

529,9701	95786,9	3	1587,8947	Brevinin-1LE	25.52-31.07
379,9952	453586,1	3,**4**,5	1516,9574	Esculentin-1R	21.09-25.67
379,9703	120134,8	4	1516,8578	Brevinin-1LE	25.52-31.07
372,99	26568,3	4	1488,9366	Esculentin-1	17.34-21.18
476,612	67414	3	1427,8204	Brevinin-2Rd	25.52-31.07
469,2321	304333,9	3	1405,6807	Esculentin-1,Esculenti	21.09-25.67
344,4791	28809,6	4	1374,8930	Esculentin-1	17.34-21.18
458,2771	3433719	2,**3**	1372,8157	Brevinin-2L	25.52-31.07
453,9502	591944,8	3	1359,8350	Esculentin-2LE	25.52-31.07
453,9501	245425,4	2,**3**	1359,8347	Esculentin-2LE	31.00-34.74
678,8959	74897,8	2	1356,7840	Brevinin-2Rd	25.52-31.07
653,3181	240440,6	2	1305,6284	Brevinin-2L	21.09-25.67
323,2129	98410,9	4	1289,8282	Esculentin-1R	21.09-25.67
416,5423	86724,8	3	1247,6113	Esculentin-1,Esculenti	21.09-25.67
596,7761	289019,1	2	1192,5444	Brevinin-2L	21.09-25.67
595,8171	76997,5	2	1190,6264	Brevinin-1LE	25.52-31.07
388,5115	10927	3	1163,5189	Brevinin-1Ra	12.10-17.63
284,1843	16886,3	4	1133,7138	Esculentin-1	17.34-21.18
373,5693	153802,5	3	1118,6923	Esculentin-2LE	25.52-31.07
555,8209	398682,6	2	1110,6340	Brevinin-1LE	31.00-34.74
350,222	88497,1	3	1048,6504	Esculentin-1R	21.09-25.67
520,3023	244773,6	2	1039,5968	Brevinin-1LE	31.00-34.74
508,311	1336972	2	1015,6142	Brevinin-2L	25.52-31.07
495,8028	2640151	1,**2**	990,5978	Esculentin-2LE	25.52-31.07
495,8027	79096,9	2	990,5976	Esculentin-2LE	31.00-34.74
483,2127	32265,6	2	965,4176	Brevinin-2L	17.34-21.18
447,6941	13242,2	2	894,3804	Brevinin-2L, Brevinin-2	12.10-17.63
412,1755	9623,4	2	823,3432	Brevinin-2L, Brevinin-2	12.10-17.63
389,2396	50605,7	1	389,2396	Brevinin-2L	17.34-21.18

ervation of Bioactive Amphibian Skin Peptides

ina
cow, Russia

Fragment of precursor peptide	mass accuracy, ppm
M+H	1
M+H	0,6
M+H	0,1
M+H	0,03
M+H	1
M+H	0,4
M+H	6,4
M+H	2,6
M+H	0,1
M+H	0,5
M+H	0,2
M+H	1
M+H	0,1
M+H	2,9
M+H	0,2
M+H	0,7
M+H	5,5
M+H	0,2
M+H	0,8
M+H	0,6
M+H	0,2
M+H	0,08
M+H	0,4
M+H	0,7
Y8-ion	0,4
y12-ion	0,5
y10-ion	3,3
y10-ion	3,3
y13-ion	0,6
y14-ion	0,3
b14+H2O-ion	0,3
b11+H2O-ion	1,3
b27+H2O-ion	0,2
y19-ion	0,1
b10+H2O-ion	0,4

M+H	1,1
M+H	4
M+H	2,9
M+H	0,4
M+H	1,4
M+H	0,4
M+H	0,4
M+H	1,1
M+H	0,8
M+H	2,4
M+H	0,2
M+H	0,1
M+H	0,7
M+H	0,9
M+H	1,1
M+H	0,1
M+H	0,4
M+H	0,04
M+H	1,1
M+H	1
M+H	0,9
M+H	1
M+H	0,2
M+H	1,3
M+H	1,2
y14-ion	0,3
y12-ion	0,5
y11-ion	0,7
y19-ion	0,1
b14+H2O-ion	0,3
b10+H2O-ion	1,7
y30-ion	0,8
y28-ion	0,5
y26-ion	0,7
y23-ion	0,5
b14+H2O-ion	0,8
b10+H2O-ion	0,4
y19-ion	0,9
y22-ion	1

M+H	4,6
M+H	0,2
M+H	4,1
M+H	0,4
M+H	0,1
M+H	0,4

M+H	2,7
M+H	0,2
M+H	0,1
M+H	0,9
M+H	1,1
M+H	0,7
M+H	0,9
M+H	0,2
M+H	0,1
M+H	0,1
M+H	0,2
M+H	1,3
M+H	1
M+H	0,2
M+H	1,1
M+H	0,9
M+H	0,1
M+H	1,5
M+H	1
y10-ion	0,4
y10-ion	0,3
y8-ion	0,6
y14-ion	0,5
y12-ion	0,8
b27+H2O-ion	0,6
b27+H2O-ion	0,3
b25+H2O-ion	0,7
b21+H2O-ion	0,5
b21+H2O-ion	0,6
y19-ion	0,5
b18+H2O-ion	0,6
b19+H2O-ion	0,5
y16-ion	0,6
b17+H2O-ion	2,2
y15-ion	0,7
y15-ion	0,4
y16-ion	0,6
y15-ion	1,1
y13-ion	0,4
y14-ion	0,4
b14+H2O-ion	0,1
b11+H2O-ion	2,4
b10+H2O-ion	0,4
b8+H2O-ion	0,3
b4+H2O-ion	0,5
y30-ion	0,8
y28-ion	1
b27+H2O-ion	1,2
y23-ion	0,7

y23-ion	0,4
y18-ion	0,7
y19-ion	1
y17-ion	0,8
b13+H2O-ion	0,9
b13+H2O-ion	0,7
b10+H2O-ion	0,4
b7+H2O-ion	0,7
y19-ion	0,9
y30-ion	2,6
y22-ion	0,6
M+H	0,2
M+H	0,5
M+H	0,9
M+H	1,3
M+H	0,5
M+H	0,9
M+H	0,7
M+H	1,2
M+H	1,8
M+H	2,4
M+H	0,7
M+H	0,3
M+H	1
M+H	1
M+H	0,9
M+H	0,9
M+H	0,9
M+H	1,1
M+H	0,7
M+H	0,9
y34-ion	0,7
y32-ion	0,9
y32-ion	0,6
y28-ion	2,6
y28-ion	0,4
y28-ion	1,3
y28-ion	0,1
y28-ion	1
y28-ion	1
y23-ion	0,3
y23-ion	0,1
y19-ion	0,5
b18+H2O-ion	0,6
y19-ion	0,5
y19-ion	0,6

y19-ion	0,8
y19-ion	0,8
y18-ion	1,1
b19+H2O-ion	1
y18-ion	0,3
y17-ion	0,2
y16-ion	0,1
y16-ion	0,6
y16-ion	0,5
b17+H2O-ion	1
y16-ion	0,9
y15-ion	0,2
y15-ion	0,9
b14+H2O-ion	0,7
y14-ion	0,8
b14+H2O-ion	0,1
y14-ion	0,8
b13+H2O-ion	0,6
y13-ion	0,9
b12+H2O-ion	1,2
b14+H2O-ion	0,3
b14+H2O-ion	0,9
b14+H2O-ion	1
b13+H2O-ion	0,4
y13-ion	0,6
y12-ion	0,6
y12-ion	0,6
y11-ion	1
b11+H2O-ion	0,5
b10+H2O-ion	0,5
b10+H2O-ion	0,5
y10-ion	0,6
y11-ion	0,6
b10+H2O-ion	2
b10+H2O-ion	1,8
b10+H2O-ion	0,9
b10+H2O-ion	0,7
b9+H2O-ion	0,5
b9+H2O-ion	0,5
b10+H2O-ion	0
b10+H2O-ion	0,6
b10+H2O-ion	0,2
b10+H2O-ion	0,4
b10+H2O-ion	0,4
b10+H2O-ion	0,6
y10-ion	0,5
y8-ion	0,7
b8+H2O-ion	1,1
b8+H2O-ion	0,4
b7+H2O-ion	0,3

b6+H2O-ion	0,3
b7+H2O-ion	0,4
b7+H2O-ion	0,4
b5+H2O-ion	0,2
b5+H2O-ion	0,5
b4+H2O-ion	0,3
M+H	1,2
M+H	0,1
M+H	0,8
M+H	0,6
M+H	0,2
M+H	1
M+H	0,9
M+H	1,1
M+H	1,1
M+H	0,1
M+H	0,4
M+H	1,1
M+H	0,9
y34-ion	0,8
y34-ion	1,1
y33-ion	0,7
y32-ion	0,7
y32-ion	0
y28-ion	0,1
y28-ion	0,1
y28-ion	1,5
y22-ion	0,6
y23-ion	0,7
y23-ion	0,6
b21+H2O-ion	1,1
y19-ion	0,3
y19-ion	1
b18+H2O-ion	1,1
b18+H2O-ion	0,1
y19-ion	0,6
y19-ion	0,6
y19-ion	0,8
y19-ion	1
b18+H2O-ion	0,5
y18-ion	1
b19+H2O-ion	0,1
y17-ion	0,9
y16-ion	0,6
y16-ion	0,6
b14+H2O-ion	0,4

y15-ion	0,9
b14+H2O-ion	0,1
y14-ion	0,8
b13+H2O-ion	0,5
b14+H2O-ion	0,8
y14-ion	0,5
b12+H2O-ion	0,9
b14+H2O-ion	0,1
b14+H2O-ion	0,6
b14+H2O-ion	0,8
b13+H2O-ion	0,4
y13-ion	0,5
b12+H2O-ion	1,6
y12-ion	0,8
y12-ion	0,4
y11-ion	0,7
y10-ion	0,7
b10+H2O-ion	1,3
b11+H2O-ion	0,8
b10+H2O-ion	0,5
b10+H2O-ion	0,9
b9+H2O-ion	0,7
b10+H2O-ion	0,4
b10+H2O-ion	0,4
b10+H2O-ion	0,6
y10-ion	0,4
y9-ion	0,4
y8-ion	0,6
b4+H2O-ion	0,5
